# Fenestrated Endovascular Aortic Aneurysm Repair (FEVAR) for Complex Thoracoabdominal and Abdominal Aortic Aneurysms: First Iranian FEVAR Series Report with Mid-Term Follow-Up

**Published:** 2018-04

**Authors:** Ali Mohammad Haji Zeinali, Kyomars Abbasi, Mahmmod Shirzad

**Affiliations:** *Tehran Heart Center, Tehran University of Medical Sciences, Tehran, Iran.*

**Keywords:** *Aorta*, *Aortic aneurysm*, *Stents*, *Iran*

## Abstract

Endovascular treatment of aortic diseases has improved in recent years. More complex thoracoabdominal and juxtarenal abdominal aortic aneurysms can now be treated with new stent grafts and techniques. Fenestrated endovascular aortic aneurysm repair (FEVAR) with fenestrated stent grafts was commenced in our center after hundred cases of endovascular aortic repair, and so far 4 serial complex cases deemed inoperable (2 juxtarenal abdominal aortic aneurysms, 1 thoracoabdominal aneurysm, and 1 thoracoabdominal pseudoaneurysm) have been treated with FEVAR. All these patients needed custom-made stent grafts, which were designed and implanted successfully under general anesthesia in the catheterization laboratory. They were followed up for more than 1 year, with a median follow-up period of 23.0 months. There were no major in-hospital or short-term complications. Only 1 patient had midterm unilateral iliac artery thrombosis, which was successfully managed interventionally. Computed tomography angiography at 1 year’s follow-up showed that the stent grafts were patent and their visceral branch cover stents had no endoleak.

## Introduction

Fenestrated endovascular aortic aneurysm repair (FEVAR) is an almost new minimally invasive technique for repairing thoracoabdominal or complex abdominal aortic aneurysms (AAAs) and has facilitated the treatment of aneurysms adjacent to visceral arteries, particularly renal arteries.^1^ This endovascular alternative to the traditional open surgical repair has helped much to prevent complications arising from the decline in the flow of the side branch arteries of the aorta.^2^ These fenestrated stent grafts are customized to each patient, and generally it takes 6 to 12 weeks to manufacture the graft.^3^ However, it is a technically challenging operation and every single patient has his/ her own characteristics and thereby difficulties during the procedure. There is, therefore, a growing need for reporting cases treated via FEVAR in order to provide sufficient evidence for endovascularists who use this method. Tehran Heart Center is the first hospital in Iran to perform endovascular aortic repair (EVAR) and FEVAR.^4^^-^^7^ We herewith introduce 4 patients with complex aortic aneurysms treated successfully with FEVAR at our specialized center, for the first time in Iran, and describe their characteristics and mid-term follow-up results.

## Case Reports

Data on 4 patients with complex aortic aneurysms who presented to our center and were treated by FEVAR are reviewed herein. The characteristics of these cases are depicted in Table 1. 

All the patients underwent FEVAR in our catheterization laboratory under general anesthesia via bilateral femoral arteriotomy and femoral sheath insertion. After an intravenous injection of 5000 IU of heparin, digital subtraction angiography for the aorta as well as the visceral and iliac arteries was performed. 

Custom-made Cook Zenith fenestrated endografts (Cook Medical Inc., Bloomington, IN, USA) were used for all the patients. They consisted of 3 components: a proximal tubular fenestrated body component, a bifurcated body graft, and iliac leg extension stent grafts. High-resolution spiral computed tomography (CT) scans and 3D workstations were employed to design the endografts. All the proximal tubular stent grafts had large holes. The patency of the endografts was checked using digital subtraction angiography at the end of the procedure. 

The final digital subtraction angiography for all the patients revealed good exclusion of the aneurysms with a normal blood flow in the celiac, superior mesenteric, renal, and iliofemoral arteries without any significant endoleak. All the patients were kept under observation in the coronary care unit (CCU). There were no major in-hospital complications, and all the patients were discharged in good physical condition.

All 4 patients were followed up regularly at the Endovascular Clinic of our center for at least 1 year for the assessment of any postprocedural complications.

**Table 1:** Patient characteristics

	Case 1	Case 2	Case 3	Case 4
				
Age (y)	48	68	71	80
				
Gender	Male	Male	Male	Male
				
Smoking	-	Cigarettes and opium	Smoker	-
				
IHD	-	Moderate IHD	CABG 10 years ago	CABG 9 years ago
				
DM	-	+	+	-
				
Renal failure	Normal Cr (1 kidney)	-	CRF (1 kidney)	-
				
COPD	-	+	+	-
				
Underlying disease	Behçet’s disease	-	-	-
				
Previous surgery	Thoracic aneurysmorrhaphy	-	CABG	CABG
				
Type of AAA	Suprarenal pseudoaneurysm	Juxtarenal AAA	Juxtarenal AAA	Thoracoabdominal aneurysm
				
Iliac arteries	Normal	Right iliac artery aneurysm	Bilateral iliac arteries stenosis	Normal
				
Proximal stent graft holes	1 (celiac)	2 (renal arteries)	2 (SMA and right renal artery)	3 (SMA and both renal arteries)
				
Concomitant IBD	-	+	-	-
				
Successful exclusion of the aneurysm	+	+	+	+
				
Postprocedural endoleaks	-	-	-	-
				
Complication within the 1^st^ year	-	Right iliac artery occlusion	-	-

IHD, Ischemic heart disease; DM, Diabetes Mellitus; Cr, Creatinine; Chronic renal failure; COPD, Chronic obstructive pulmonary disease; CABG, Coronary artery bypass graft; AAA, Abdominal aortic aneurysm; SMA, Spinal muscular atrophy

***Case #1***

A 48-year-old man, who was a known case of Behçet’s disease and had a single right kidney, presented with pseudoaneurysm of the aorta near a stenotic celiac truck and the superior mesenteric artery just below a previous thoracic stent graft. The patient had a history of descending thoracic aorta aneurysm, which had been treated via aneurysmorrhaphy 6 years previously, and the recurrence of the pseudoaneurysm in the same place, which had been treated with a thoracic stent graft 3 years earlier. The consulting surgeon recommended a hybrid FEVAR procedure‌ for this patient given his history of Behçet’s disease and 2 recent operations. 

**Figure 1:**
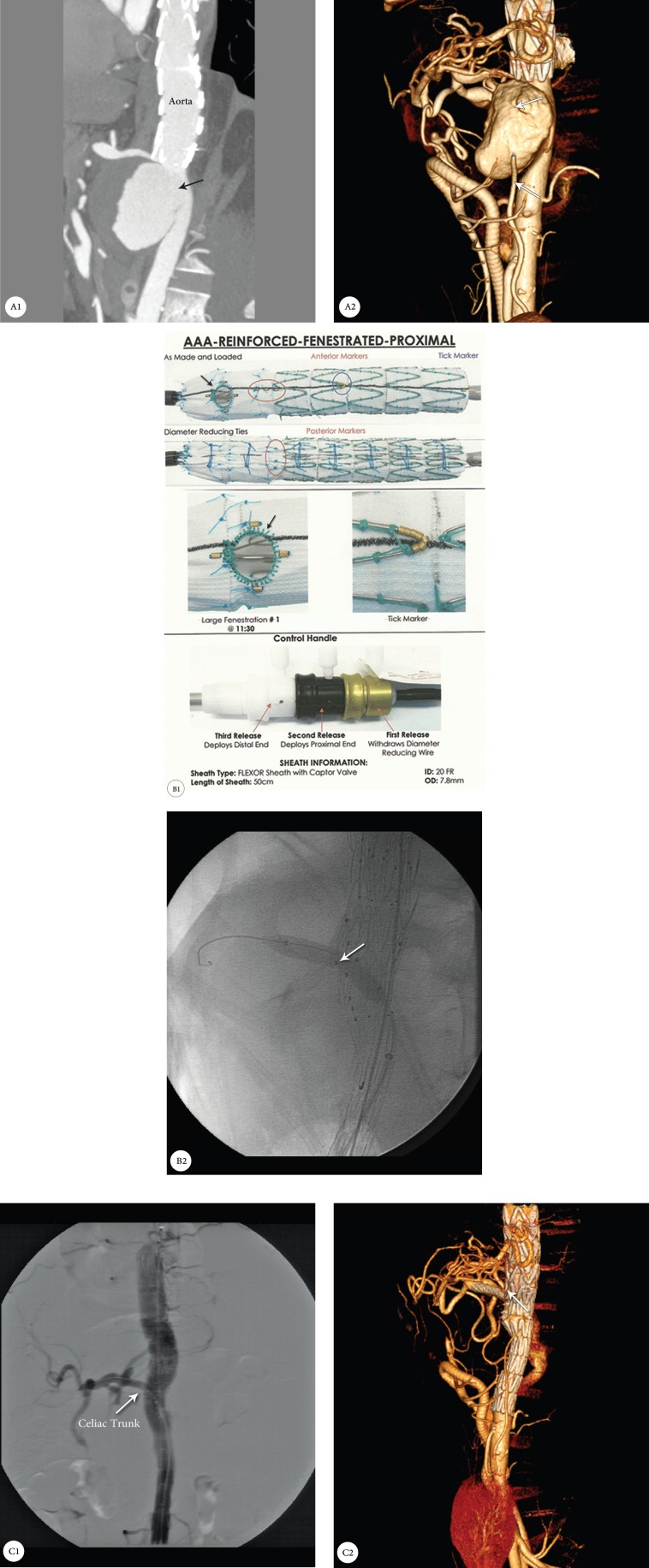
A1 & A2: Computed tomography angiography of the thoracoabdominal aorta, showing a pseudoaneurysm (arrows); B1: Location of the hole in the custom-made 1-hole stent graft (arrow); B2: Fluoroscopy in the lateral view, showing balloon inflation in the celiac trunk from the stent graft hole (arrows); C1 & C2: Postprocedural computed tomography angiography, showing fenestrated endovascular aortic aneurysm repair with the exclusion of the pseudoaneurysm and the perfusion of the celiac trunk (arrows).

In our center, the hybrid FEVAR operation was performed because of the complexity of the pseudoaneurysm. After the surgical connection of the left renal artery and superior mesenteric artery to the iliac artery, a custom-made 1-hole tubular stent graft was implanted in the thoracoabdominal area and the celiac artery was stented with an Atrium V_12_ cover stent (Figure 1). Our anatomical problem was the severe stenosis of the origin of the celiac trunk, prompting us to first perform balloon angioplasty and then implant the fenestrated stent graft.

***Case #2***

A 68-year-old man, who was a smoker and opium abuser, presented with a symptomatic large AAA adjacent to the renal arteries. The patient’s AAA was extended to the right iliac artery, rendering him high risk for open surgery due to concomitant chronic obstructive pulmonary disease (COPD) and a low ejection fraction (30%). 

He underwent FEVAR with the implantation of a proximal fenestrated tubular stent graft making sure of the good apposition of the gold markers around the fenestrated area. (These markers are very important for the correct positioning of each hole in front of the ostium of each artery.) Thereafter, both renal arteries were cannulated with a Super Stiff guide wire (0.035 inch), stented with balloon-expandable Atrium V_12_ cover stents, and post-dilated and flared for good apposition to the main tubular stent grafts. Subsequently, routine extending of the stent grafts for the abdominal and iliac arteries was done. 

**Figure 2:**
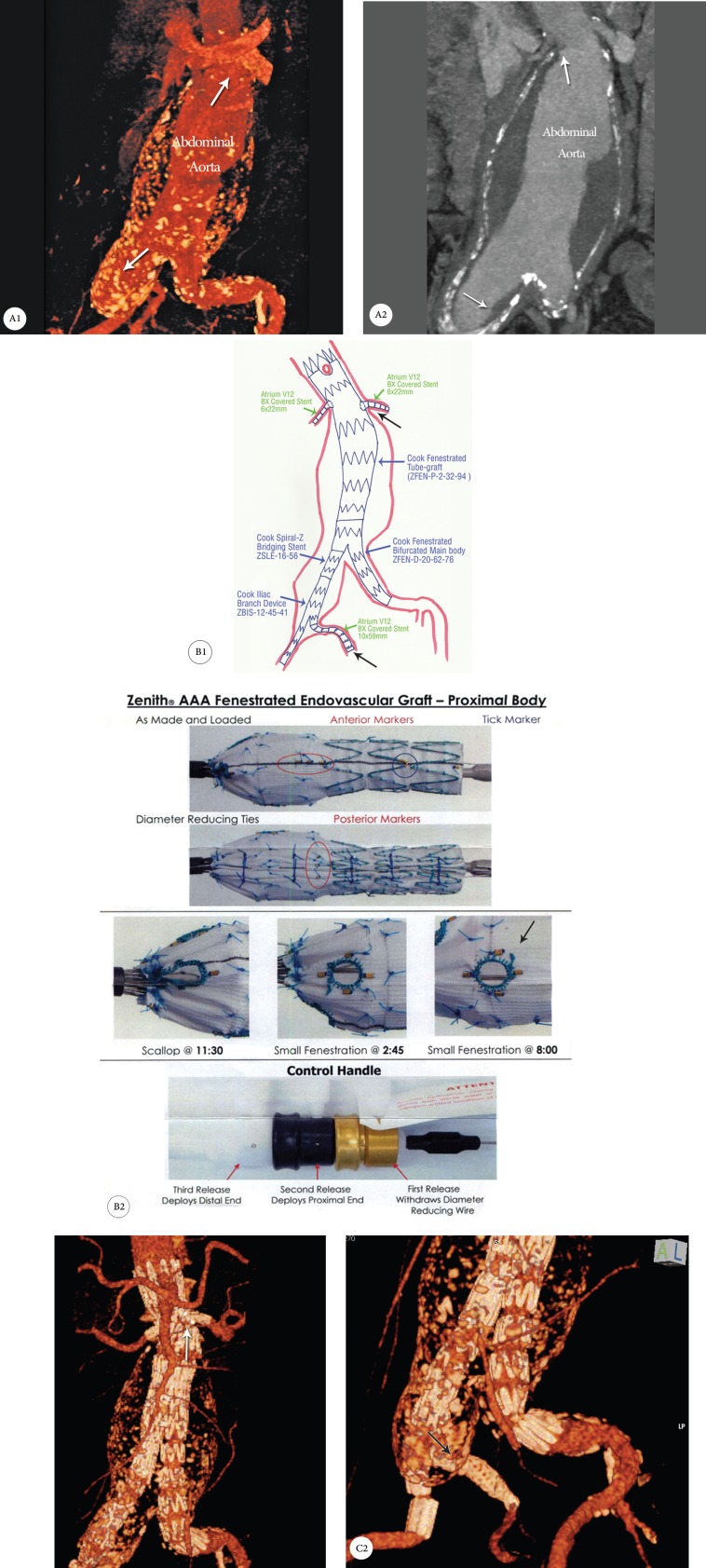
A1 & A2: Computed tomography angiography, showing a juxtarenal abdominal aortic aneurysm with a large right iliac aneurysm (white arrows); B1 & B2: Schematic location of the fenestrated custom-made stent graft with the iliac branch device; C1 & C2: Computed tomography angiography following fenestrated endovascular aortic aneurysm repair, showing the exclusion of the aneurysm and a patent right internal iliac artery branch (black arrow) and both renal arteries (white arrow).

Because of a large right common iliac aneurysm, we also used an iliac branch device so as to save the right internal iliac artery flow (Figure 2). This procedure was technically challenging and needed approaching both femoral arteries. After the main body of the iliac branch device was placed on the right iliac artery, the prepared guide wire was snared from the left femoral sheath. Over this wire, the right internal artery was cannulated and stented with an Atrium V_12_ cover stent. Finally the iliac branch device was implanted.

***Case #3***

**Figure 3:**
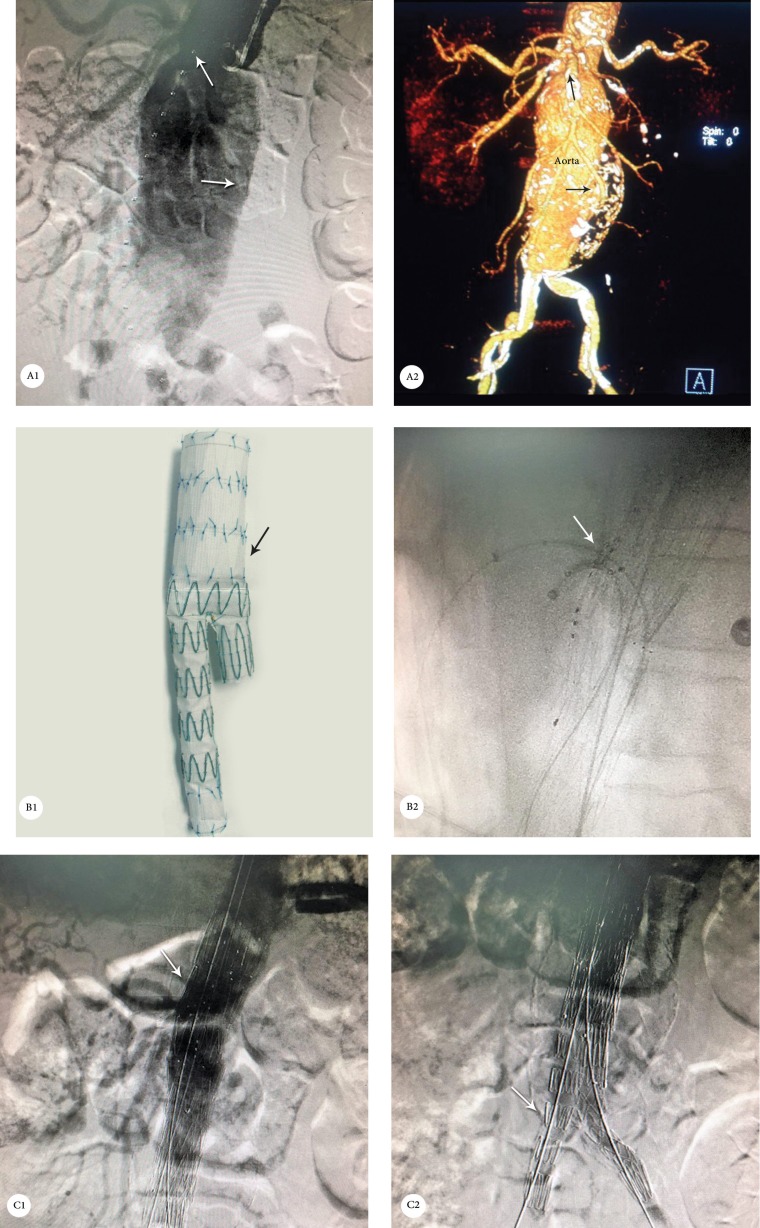
A1 & A2: Abdominal computed tomography angiography, showing a juxtarenal aortic aneurysm (arrows); B1: Main body of the abdominal aortic stent graft; B2: Fluoroscopy, showing the cannulation of the holes of the custom-made fenestrated stent graft; C1: Digital subtraction angiography following fenestrated endovascular aortic aneurysm repair, showing the successful exclusion of the abdominal aorta aneurysm (arrow); C2: Digital subtraction angiography following fenestrated endovascular aortic aneurysm repair, showing patent iliac artery stent grafts (arrow).

A 71-year-old man with a history of hypertension, chronic renal failure with a single kidney, COPD due to smoking, and history of coronary artery bypass graft surgery 10 years previously was referred to our center because of abdominal pain. CT angiography revealed a juxtarenal AAA and bilateral stenosis of the iliac arteries. The patient underwent FEVAR with the implantation of a proximal stent graft that had holes for the superior mesenteric artery and 1 renal artery. Both arteries were cannulated. Thereafter, cover stenting and routine post-dilation with a CODA balloon were performed for the overlapping area and the proximal and distal necks. The stenosis of the iliac arteries was improved with the balloon post-dilation of the stent graft in the stenotic area (Figure 3).

***Case #4***

**Figure 4:**
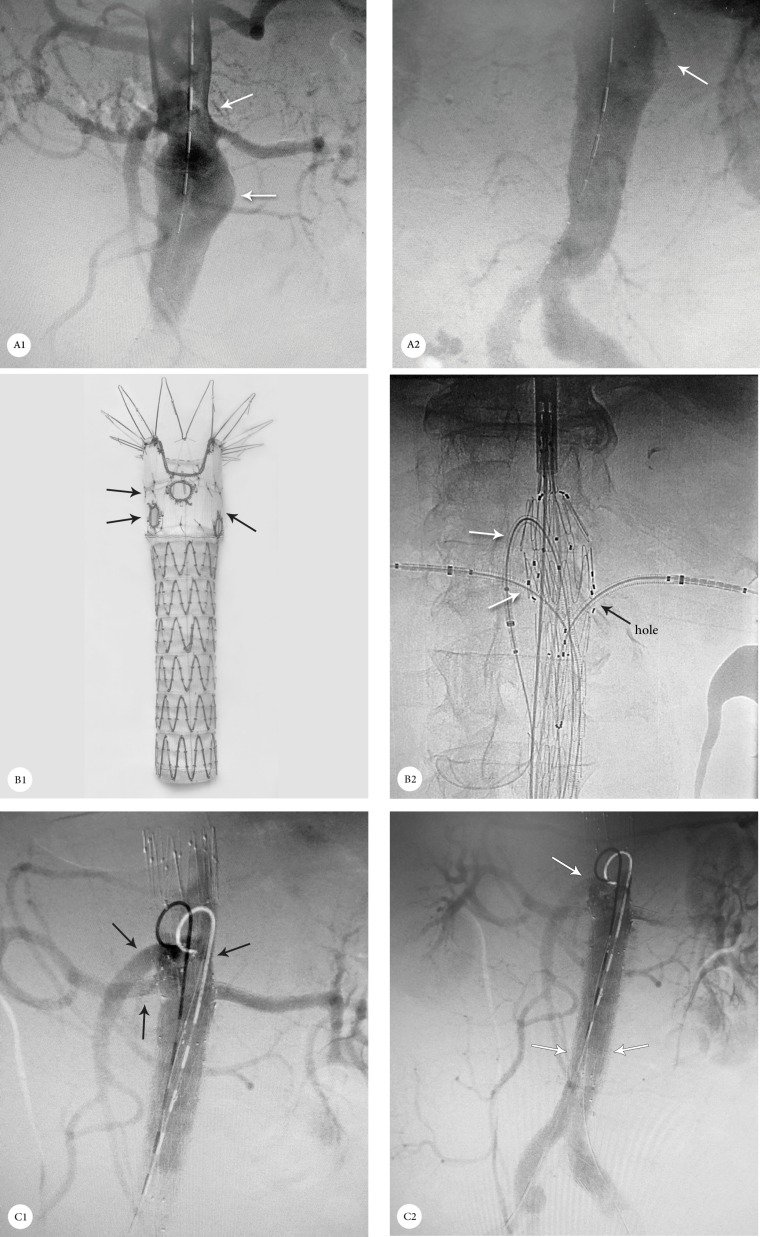
A1 & A2: Computed tomography angiography, showing a thoracoabdominal aneurysm (arrows); B1: Location of the holes (arrows) in the custom-made 3-hole stent graft; B2: Fluoroscopy in the anterior posterior view, showing the successful cannulation from the 3 holes of the stent graft; C1 & C2: Digital subtraction angiography following fenestrated endovascular aortic aneurysm repair with the exclusion of the aneurysm (white arrows) and perfusion of the celiac artery, superior mesenteric artery, and both renal arteries (black arrows).

An 80-year-old man with a thoracoabdominal aneurysm that involved the celiac trunk, superior mesenteric artery, and both renal arteries was referred to our center. Because of the patient’s low ejection fraction and previous coronary artery bypass graft surgery, open surgical repair was very high risk. He underwent totally standard FEVAR with a proximal tubular stent graft that had 1 scallop for the celiac trunk and 3 holes for the superior mesenteric artery and both renal arteries. All the side branches were stented with an Atrium V_12 _cover stent. Distal abdominal stent grafting with both iliac artery extensions was done based on standard FEVAR. After balloon post-dilation, digital subtraction angiography revealed successful exclusion of the aneurysm without any endoleak or side-branch compromise (Figure 4).

## Discussion

Development of FEVAR has conferred a suitable method for the endovascular treatment of complex thoracoabdominal or juxtarenal AAAs with unsuitable proximal necks. Despite the high professional and technical skills required for utilizing these graft stents, the reported results are noteworthy. However, due to the limited number of procedures and variety in patients’ conditions and clinical settings, reporting the outcome of every procedure is still demanding. 

Our study reports the first FEVAR case series in our country and supports the eventless use of FEVAR for patients with complex thoracoabdominal or juxtarenal AAAs. All our patients were followed up for more than 1 year, with a median follow-up period of 23.0 months. It is worthy of note that none of our patients had rupture or conversion to surgery, and nor did they require dialysis or expire from target-vessel occlusion. Nonetheless, only 1 patient required secondary intervention owing to right external iliac artery occlusion, which was successful. 

In the follow-up period, the second case (Case#2) presented some ischemic symptoms of the right lower extremity in the fourth month. Our evaluation showed the occlusion the right iliac artery stent graft due to thrombosis in the iliac artery tortuosity. The patient underwent successful balloon angioplasty on the right iliac artery.

We successfully performed the FEVAR procedure in all 4 patients and observed no major clinical or technical complications such as the occlusion or dislocation of the stent grafts. Our final digital subtraction angiography revealed good exclusion of the aneurysms with a good flow in the celiac, superior mesenteric, renal, and bilateral iliac arteries. The patients were transferred to the CCU and were closely monitored. The mean length of hospital stay was 5.75±2.87 days (range=4–10 d), based on the general condition and comorbidities of the patients. 

Our patients experienced no other problems within their 1-year follow-up. Clinical examinations, laboratory examinations (especially for the kidney function), and CT angiography of the aorta were satisfactory at the end of the first year for all 4 patients.

Case selection in FEVAR procedures depends highly on the anatomy of the target vessel as the manufacturing of a custom-made graft has its own engineering limitations.^8^ Since larger fenestrations can ensure better patency,^9^ we used larger fenestrated (6×8 mm) endografts to guarantee the success of the procedure. 

Similar to any other intervention, the use of FEVAR is not without complications. Most of the relevant studies have reported mid- to long-term results of the procedures. The reported complications for FEVAR include the stenosis and ischemia of side branches (particularly mesenteric and renal arteries) and their resulting adverse events, tilting, kinking or fracture of stents, enlargement of aneurysm sacs, rupture of aneurysms, endoleaks, stent thrombosis, and access-site complications like hematoma.^1^^, ^^10^^, ^^11^ Muhs et al.^12^ studied 38 FEVAR cases with a median follow-up period of 25 months and reported a 1-year mortality rate of 13%. In the same study, 3 vessels were lost postoperatively and the cumulative visceral branch patency was 92% at 46 months. All the other incidents requiring secondary intervention happened in the first year of the procedure. In another study, endoleaks were the most common complication.^13^ The only complication observed in our study was late stent thrombosis in the external iliac leg of the stent. In a propensity-matched study, Raux et al.^14^ evaluated 59 FEVAR procedures that had been done in a period of 11 years and suggested that FEVAR was allied to high risk for preoperative mortality and morbidity compared with open surgical repair. The authors, however, recommended further widespread use for comparison. 

The present study was a single-center study with a limited number of cases; nevertheless, this is the first experiences of FEVAR with a mid-term follow-up in Iran. The price of custom-made stent grafts is another salient limitation to the use of FEVAR for some patients.

## Conclusion

The FEVAR procedure is an acceptable alternative to open surgical repair in high-risk patients due to comorbidities. Our experience with FEVAR in limited challenging cases was successful. Given that every case of FEVAR requires a specific plan, we hope that the results of our study will contribute to collective experience the world over. 

## Notes:

***This paper should be cited as: ***Haji Zeinali AM, Abbasi K, Shirzad M. Fenestrated Endovascular Aortic Aneurysm Repair (FEVAR) for Complex Thoracoabdominal and Abdominal Aortic Aneurysms: First Iranian FEVAR Series Report with Mid-Term Follow-Up. J Teh Univ Heart Ctr 2018;13(2):88-98.
